# The Role of Coffee Microbiomes in Pathogen Resistance Across Varieties and Ecological Niches

**DOI:** 10.3390/microorganisms13081909

**Published:** 2025-08-15

**Authors:** Yihong Wu, Xiu Zhao, Zuquan Wang, Xuejun Li, Xuesong Zhang, Chun Xie, Huabo Du, Kuaile Jiang, Peng Qu, Chuanli Zhang

**Affiliations:** 1 College of Tropical Crops, Yunnan Agricultural University, Pu’er 665001, China; 2024240849@stu.ynau.edu.cn (Y.W.); zhaox@ynau.edu.cn (X.Z.); 2023240805@stu.ynau.edu.cn (Z.W.); 2003056@ynau.edu.cn (X.L.); 2020240160@stu.ynau.edu.cn (X.Z.); 2008077@ynau.edu.cn (C.X.); 2000047@ynau.edu.cn (H.D.); 2007070@ynau.edu.cn (K.J.); 2 Yunnan Provincal Key Laboratory of Coffee, Pu’er 665001, China

**Keywords:** coffee plant microbiome, rust-resistant coffee varieties, ecological niche specialization, co-occurrence network analysis, microbial community structure

## Abstract

The plant microbiome plays a role in pathogen defense, but its role in different resistant varieties and ecological niches remains unclear. This study used 16S rRNA and ITS sequencing to investigate microbial communities and interactions in disease-resistant (PT) and susceptible (Bourbon) coffee varieties of five ecological niches: leaves, fruits, roots, rhizosphere soil, and non-rhizosphere soil. We found that the microbial communities differed significantly between the two varieties. The resistant variety was enriched in beneficial bacteria from the Actinobacteriota phylum and a stable, modular microbial network dominated by saprotrophic fungi. In contrast, the susceptible variety had a higher abundance of opportunistic pathogens and stress-indicator fungi, including *Neurospora* spp., which were more prominent in the rhizosphere and non-rhizosphere soils. These networks were fragile and dominated by pathotrophic fungi, reflecting ecological imbalance. Our findings show that plant disease resistance is influenced not only by host genetics but also by co-evolutionary interactions with the microbiome. These insights provide a foundation for developing targeted biocontrol strategies to manage plant-associated microbial communities.

## 1. Introduction

Coffee (*Coffea* spp.), belonging to the Rubiaceae family, is primarily cultivated in two species: Arabica (*Coffea arabica*) and Robusta (*Coffea canephora*) [1]. It is the third-largest agricultural export in Yunnan Province, China, with 118,000 hectares under cultivation and a total output of 139,000 tons. Yunnan accounts for over 95% of both the cultivated area and total coffee output in China [2]. Arabica coffee, a perennial species, is particularly susceptible to various diseases. Climate change exacerbates the spread of major coffee diseases, significantly impacting yield and quality [3,4,5]. Among these, coffee leaf rust (CLR), caused by the fungus *Hemileia vastatrix*, is the most destructive, leading to substantial economic losses and presenting significant research challenges due to the obligate endoparasitic nature of the fungus, which cannot thrive in pure cultures or artificial media [6]. CLR primarily infects coffee leaves, causing defoliation and impacting yield and quality [7]. Therefore, understanding the microbial composition and structure of coffee plants exhibiting diverse disease resistance phenotypes is crucial for breeding CLR-resistant coffee varieties.

Bacteria and fungi are the dominant components of plant microbial communities [8]. Microbial interactions within different hosts and ecosystems are crucial, influencing evolutionary and co-evolutionary dynamics in microbiomes [8]. Rhizosphere microorganisms, including bacteria, fungi, and other soil-dwelling microbes, play a key role in mediating plant–soil interactions, significantly influencing soil organic matter decomposition and nutrient cycling [9,10,11]. These microbial processes are crucial for converting complex organic compounds into simpler, bioavailable forms, thus improving crop yields and enhancing plant resilience to both biotic and abiotic stresses [12,13]. Moreover, the dynamics of these interactions are shaped by evolutionary processes, where microbial communities adapt to environmental pressures, promoting long-term ecological stability and influencing plant health.

However, research on microbial communities in coffee’s other ecological niches remains limited. While plant and animal ecological niches have been widely studied, microbial niches are less explored [14]. The 16S rDNA amplicon sequencing technique has become a cornerstone for analyzing microbial composition and structure in environmental samples [15], while ITS sequencing aids in identifying fungi [15,16,17]. High-throughput sequencing, especially Illumina sequencing, is now the preferred method due to its higher accuracy and throughput [18,19,20,21]. This technique has enabled in-depth studies of microbial community composition and function, including those of disease-resistant and susceptible microbial communities, identifying core microorganisms, and predicting functions [22].

Recent research on plant disease resistance has shifted from single pathogen recognition to exploring multi-level interactions involving plants, microorganisms, and their environment [23]. Microbial communities play a critical role in protecting plants from diseases [24]. The subset of these communities tightly associated with a specific plant species or genotype, independent of soil and environmental conditions, constitutes the core plant microbiome [25]. These microorganisms are thought to provide long-term benefits to the plant, often through mechanisms such as pathogen inhibition, nutrient cycling, and immune modulation. Studies show that different plant varieties harbor distinct microbial communities, even in the same environment, with host-selection mechanisms significantly influencing disease resistance [26]. Disease-resistant plants tend to have higher microbial diversity and distinct functional groups, with genera like *Pseudomonas*, *Bacillus*, *Streptomyces*, and non-pathogenic *Trichoderma* and *Fusarium* strains exhibiting disease-suppressive properties [27]. Additionally, differences in microbial ecological niches between above-ground and below-ground tissues impact disease resistance [28,29].

Despite these insights, research on microbial interactions in coffee plants remains underdeveloped, particularly concerning perennial crops like coffee. While coffee microbiology has largely focused on fermentation and metabolic potential, little is known about the relationship between disease resistance and symbiotic microorganisms [30,31]. This study aims to systematically characterize the bacterial and fungal community structures in both disease-resistant and susceptible *Coffea arabica* plants. This research will span five distinct ecological niches: non-rhizosphere soil, rhizosphere soil, roots, leaves, and fruits. By employing high-throughput sequencing technology, this study seeks to uncover how these microbial communities contribute to disease resistance, and it will explore the potential for establishing a microbe-driven ecological regulatory system to improve the sustainability of coffee cultivation practices.

## 2. Materials and Methods

### 2.1. Experimental Design and Plant Materials

The present study selected two *Coffea arabica* L. varieties with distinct disease resistance profiles for investigation: the Bourbon variety (susceptible to coffee leaf rust) [32] and the PT variety (resistant to coffee leaf rust) [33]. The selection of PT (Progeny86) and Bourbon varieties for this study was based on their distinct disease resistance profiles, as documented in prior research [34,35]. While Bourbon (including its Typica variant) is highly susceptible to coffee leaf rust (*Hemileia vastatrix*) and lacks significant immunity, PT (Progeny86) has demonstrated robust resistance to this pathogen [36,37]. In addition to the distinct disease resistance profiles, other phenotypic differences between the Bourbon and PT cultivars include variations in leaf morphology, fruit size, and yield potential. Bourbon typically exhibits broader leaves, stronger branches with a more vertical growth habit, and higher yield potential, whereas PT displays strong rust resistance, larger fruit, but lower yield stability, making it a preferred choice for farmers in specific regions [36,37].

Sampling was conducted at the Coffee Germplasm Resource Nursery of the Tropical Crop College, Yunnan Agricultural University, located in Pu’er City, Yunnan Province, China (geographical coordinates: 22.747466°N, 101.032922°E, alt 1 470 m asl). The four-year-old Coffea arabica plants underwent consistent management practices—including pruning, weed control, and harvesting—throughout the pre-sampling growing season. Fertilization regimes (organic fertilizer: 1000 kg/ha; compound fertilizer [15% N-P-K]: 5000 kg/ha) were uniformly administered to both cultivars. Soil conditions, characterized by a sandy-clay texture and acidity (pH 5.01 ± 0.04), were consistent across cultivar plots, ensuring experimental comparability.

### 2.2. Sampling Collection

Samples were collected from two distinct coffee varieties cultivated in a designated planting area. Three healthy adult coffee plants, exhibiting no visible signs of pests or diseases, were randomly selected from each variety as independent biological replicates. To capture a representative range of ecological niches, samples were collected from five distinct zones: fruit (Fruit), leaf (Leaf), absorbent root (Root), rhizosphere soil (Rhizosphere Soil), and non-rhizosphere soil (Bulk Soil). Rhizosphere soil and root samples were collected from the upper 0–20 cm of soil, at a distance of 1 m from the tree trunks, ensuring that fertilized zones were avoided. A sterile stainless-steel trowel was employed to carefully excavate soil containing the target root system, which was then placed intact into a sterile self-sealing bag. To isolate rhizosphere soil, soil was separated from the root system by gently brushing off the loosely adhered soil particles in a controlled laboratory environment. Non-rhizosphere (bulk) soil samples were collected from root-free inter-row zones, where the soil was not directly influenced by the root system. Visible stones and plant debris were removed from the bulk soil samples before placing them into sterile self-sealing bags. Fruit and leaf samples were collected using sterile surgical scissors and immediately placed into sterile self-sealing bags. Root and rhizosphere soil samples were processed in a similar manner. All sample bags were promptly placed in an insulated sampling box containing ice packs, and the samples were transported to the laboratory at temperatures below 4 °C, within two hours of collection. Upon arrival at the laboratory, fruit, leaf, and absorbent root samples were washed with sterile water and dried using sterile filter paper. The rhizosphere soil was carefully separated from clumped soil, and tightly adhered soil particles were gently removed from the root system using a sterile brush. All samples were stored at −80 °C in an ultra-low-temperature freezer for long-term preservation until downstream molecular biological analyses, including DNA extraction. A total of 30 samples were collected, representing 2 coffee varieties, 3 biological replicates, and 5 ecological niches.

### 2.3. DNA Extraction and High-Throughput Sequencing

Total DNA was extracted from each sample using the CTAB method to ensure the highest quality of DNA. Polymerase chain reaction (PCR) amplification was performed with 16S rRNA (primers 515F/806R targeting the V4 region) and internal transcribed spacer (ITS) (primers ITS5-1737F/ITS2-2043R) primers to analyze bacterial and fungal communities, respectively. Library construction was carried out using the TruSeq^®^ DNA PCR-Free Sample Preparation Kit. The constructed libraries were then quantified using Qubit and Q-PCR. Following library qualification, sequencing was performed using the NovaSeq6000 platform. Bacterial Taxonomy Classification: For bacterial taxonomy, the Silva Database (version 138, http://www.arb-silva.de/, accessed on 1 May 2025) was used to annotate taxonomic information based on the Mothur algorithm [36]. Fungal Taxonomy Classification: For fungal taxonomy, the Unite Database (version 9, updated 29 November 2022, https://unite.ut.ee/, accessed on 5 May 2025) was used to annotate taxonomic information based on the BLAST algorithm [37].

### 2.4. DNA Extraction and High-Throughput Sequencing Protocols

The raw data were first divided into individual sample data based on the barcode sequences and PCR amplification primer sequences. Both the barcode and primer sequences were then removed. The fastp software (v0.22.0, available at (https://github.com/OpenGene/fastp, accessed on 10 May 2025)) was used to filter the raw reads and obtain high-quality reads. The filtering process followed these steps: first, adapter sequences were automatically detected and removed; second, reads with 15 or more N bases were removed; third, reads in which low-quality bases (quality value ≤ 20) accounted for over 50% of the sequence were discarded; fourth, reads with an average quality below 20 in a 4-base window were deleted; fifth, polyG sequences were removed from the end; and sixth, reads shorter than 150 bp were excluded. After filtering, high-quality paired-end reads were assembled using FLASH (v1.2.11, available at (http://ccb.jhu.edu/software/FLASH/, accessed on 10 May 2025)) to generate clean tag data (Clean Tags). The clean tag sequences were then clustered into Operational Taxonomic Units (OTUs) using VSEARCH (v2.22.1, available at (https://github.com/torognes/vsearch/, accessed on 10 May 2025)), with a 97% similarity cut-off. VSEARCH was also used to detect and remove chimeric sequences, ensuring the generation of valid data (Effective Tags).

### 2.5. Alpha and Beta Diversity Analyses of Microbial Communities

In accordance with the standardized OTU table, the vegan package (v2.6-10) was used in R (v4.3.2) to calculate alpha diversity indices of the microbial community for each sample, including species richness (Observed OTUs), Shannon diversity index (Shannon index), and phylogenetic diversity (Faith’s PD). In this study, we define “shared” OTUs as those that appear in at least one of the three biological replicates within each ecological niche. Specifically, an OTU is considered to be present in a given ecological niche if it is detected in any one of the three biological replicates of that niche. This approach allows for flexibility in detecting the presence of OTUs across the ecological niches while accounting for potential variability between replicates. This method ensures that we capture the diversity of OTUs within each ecological niche without requiring all replicates to contain the same OTU. The ggplot2 package was used to plot dilution curves, rank-abundance curves, and species accumulation curves. A significant difference in alpha diversity indices between groups was found, and this was assessed using non-parametric Kruskal–Wallis tests for fungal diversity and Tukey’s HSD tests for bacterial diversity. All statistical tests were performed in R. The assessment of overall differences in microbial community composition among samples was conducted by calculating a community dissimilarity distance matrix based on standardized OTU tables, mainly using Bray–Curtis dissimilarity and weighted UniFrac distance. Principal coordinate analysis (PCoA) was performed on the distance matrix using the stats::cmdscale() function, and group differences in community composition were tested using permutation multivariate analysis of variance (PERMANOVA). Non-metric multidimensional scaling (NMDS) was performed using the vegan::metaMDS() function, and intergroup differences were tested using similarity analysis (ANOSIM). The sample clustering was based on the Bray–Curtis distance matrix, with the unweighted group mean average method (UPGMA) used to construct a hierarchical clustering tree of samples.

### 2.6. Identification of Significantly Different Microbial Communities

To identify microbial communities exhibiting significant abundance differences between subgroups, this study adopted an integrated linear discriminant analysis effect size (LEfSe) and random forest (RF) analysis strategy. LEfSe analysis was performed using R, using the non-parametric Kruskal–Wallis test to identify significant differences among groups, followed by the Wilcoxon rank-sum test to assess pairwise group differences. Subsequently, linear discriminant analysis (LDA) was used to estimate the magnitude of each taxon’s contribution to group differentiation. Random forest analysis was performed using the randomForest package in R to build a classification model, with the mean decrease accuracy (MDA) metric used to assess the importance of microbial taxa. The RF model was constructed with 500 trees, and model performance was validated using 10-fold cross-validation. The optimal number of variables selected at each split (mtry) was determined through tuning based on classification accuracy. The results of the RF analysis were then combined with those of the LEfSe analysis to identify microbial taxa exhibiting significant differences. The final results were visualized using the ggplot2 and patchwork packages [38,39].

### 2.7. Microbial Co-Occurrence Network Analysis

The construction of microbial co-occurrence networks is facilitated by the R package ggClusterNet (v2.0), using standardized microbial abundance tables at a designated taxonomic level. The association between microbial taxa should be calculated using SparCC or Spearman rank correlation, and significantly associated edges should be screened. The network structure is assessed by calculating topological properties (e.g., average degree, clustering coefficient) and evaluating the network’s stability under random node failure using robustness analysis [40]. Using the functionality of ggClusterNet, a comparison was made of network properties across different groups. Multi-factor analysis was employed to explore the influence of specific factors on the network structure. Network visualization was conducted using the ggraph and igraph packages, with nodes representing abundance or connectivity, and edges indicating the strength of associations.

### 2.8. Data Statistics

The initial dataset was organized using Microsoft Excel 2021. All statistical analyses and advanced visualizations were performed in the R (v4.3.2) environment, primarily relying on the tidyverse, vegan, phyloseq, randomForest, and ggClusterNet packages. The final charts were generated using the ggplot2 and patchwork packages.

## 3. Results

### 3.1. Microbial Sequencing and De Novo Assembly of Resistance Varieties of Coffea arabica Across Diverse Ecological Niches

**Figure 1:**
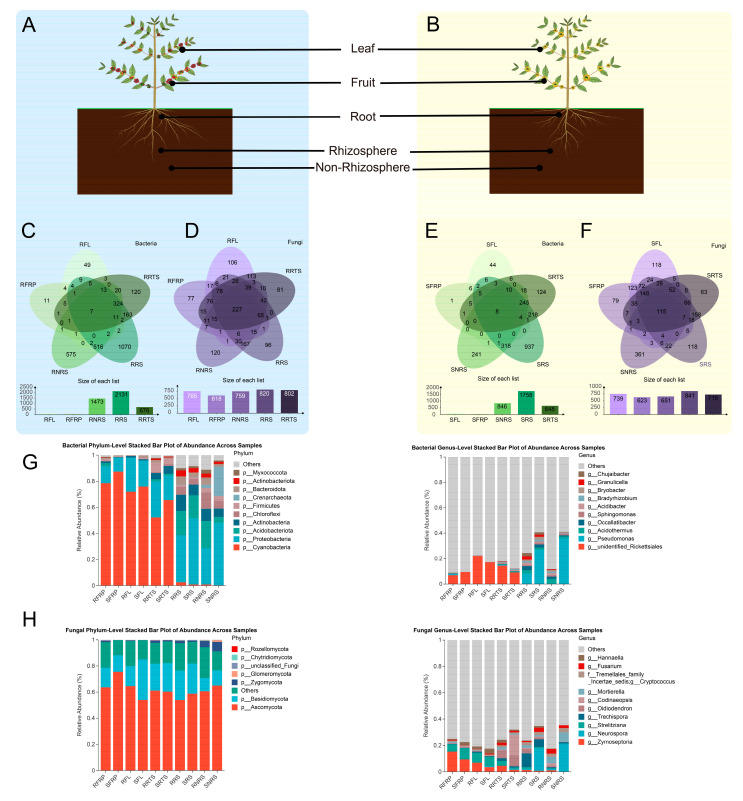
Composition and distribution of microbial communities in disease-resistant (PT) and disease-susceptible (Bourbon) coffee varieties across ecological niches: leaves (Lf), fruits (Fr), roots (Rt), rhizosphere soil (RS), and non-rhizosphere soil (NRS). (**A**,**B**) Plant schematics of PT and Bourbon varieties. (**C**) Bacterial communities in PT showing beneficial enrichment in Rt/RS. (**D**) Fungal communities in PT with increased Ascomycota in Rt/RS. (**E**) Bacterial communities in Bourbon with pathogen-enriched genera in RS/NRS. (**F**) Fungal communities in Bourbon with higher pathogen abundance in RS/NRS. (**G**,**H**) Relative abundance of bacterial (**G**) and fungal (**H**) communities at phylum/genus levels. Venn diagrams (not shown) indicate significant microbial differences between varieties, particularly in soils.

A total of 30 bacterial samples were collected, representing 2 coffee varieties, 5 ecological niches, and 3 biological replicates from both the susceptible Bourbon variety (designated as Susceptible, S) and the resistant PT variety (designated as Resistant, R). These samples were collected from five ecological niches: ripe fruit (FRP), fruit leaf (FL), root (RTS), rhizosphere soil (RS), and bulk soil (NRS), along with the susceptible Bourbon variety (S) and the resistant PT variety (R). High-throughput sequencing analysis of bacterial 16S rRNA genes and fungal ITS regions was conducted on these samples. The dilution curves showed a tendency to plateau (Figure S1), suggesting that the sequencing depth was sufficient to capture the majority of microbial species in the samples. After rigorous quality control and sequence assembly, bacterial sequencing produced 2,474,095 raw reads, which were filtered to yield 2,342,229 high-quality reads. In total, 2,054,391 sequences were used for clustering, with an average of 78,074 sequences per sample and an average length of 123 base pairs. Based on these sequences, 10,522 bacterial operational taxonomic units (OTUs) were identified. Fungal sequencing produced 2,561,643 raw reads, which underwent quality control to yield 2,477,143 high-quality reads. After clustering, 2,285,458 sequences were used, with an average of 82,571 sequences per sample and an average length of 716 base pairs, resulting in 4974 fungal operational taxonomic units (OTUs). Detailed sequencing data statistics are provided in Tables S1 and S3. The number of microbial species (OTUs) varied among different ecological niches and variety combinations (Figure 1C–F, and Tables S2 and S4). The total number of OTUs in soil ecological niches (rhizosphere soil (RS) and non-rhizosphere soil (NRS)) was significantly higher than in plant tissue niches (root (RTS), leaf (FL), and fruit (FRP)). The number of species in plant tissue ecological niches (root, leaf, fruit) was generally low. Notably, in the leaf (FL) and fruit (FRP) ecological niches, the number of bacterial OTUs was below 500 in both susceptible (S) and resistant (R) varieties (Figure 1C,E). The non-rhizosphere soil (NRS) ecological niche exhibited the highest species richness. Notably, the bacterial OTU richness in the non-rhizosphere soil (NRS) of resistant varieties was higher than in the non-rhizosphere soil (NRS) of susceptible varieties, with a difference of 627 OTUs (Figure 1C,E). Among fungi, the SNRS niche exhibited the highest number of unique OTUs (361), while among bacteria, the SRS niche exhibited the highest number of unique OTUs (937). The number of species in the rhizosphere (RS) niche was higher in both bacteria and fungi compared to plant tissues and soil. To identify microbial groups consistently present across all niches in resistant varieties, an analysis of core shared OTUs was conducted (Figure 1C–F). The Venn diagrams (Figure 1C–F) illustrate the shared OTUs between ecological niches across three biological replicates. In these diagrams, an OTU is considered “shared” within an ecological niche if it is present in at least one of the three biological replicates of that niche. This threshold ensures that OTUs detected in any replicate are included in the analysis, allowing for a broader assessment of microbial diversity within each ecological niche. The number of bacterial core shared OTUs was extremely low and similar across varieties. A total of seven bacterial OTUs were identified in all ecological niches of the disease-resistant variety (R), whereas eight bacterial OTUs were detected in the disease-susceptible variety (S) (Figure 1C,E). This finding suggests that the core bacterial communities present in all ecological niches are extremely rare, irrespective of the level of variety resistance. In contrast, the fungal core exhibited a comparatively higher number of OTUs, with significant differences observed between species. The number of core fungal OTUs identified in the resistant variety (R) amounted to 227, nearly double the number observed in the susceptible variety (S), which was 115 (Figure 1D,F). These “shared” core OTUs were identified as described in the Methods section, based on the presence of specific fungal taxa across all samples within each variety. This finding indicates that resistant varieties may possess a core fungal community that is more extensively distributed across various plant parts (leaves, fruits, roots) and their surrounding soil (rhizosphere, non-rhizosphere).

### 3.2. Alpha Diversity Analysis Reveals the Joint Influence of Ecological Niches and Species on Microbial Community Diversity

**Figure 2:**
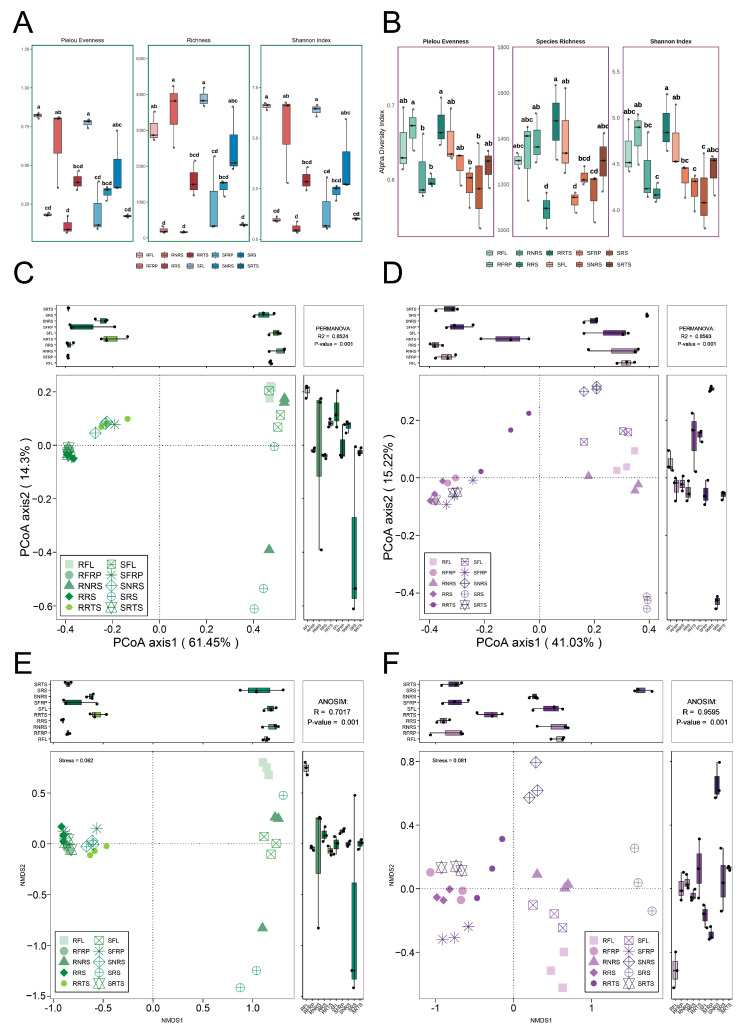
Microbial community diversity analysis: comparison between disease-resistant PT varieties and susceptible Bourbon varieties of *Coffea arabica* species. Note: Analysis of microbial diversity differences between disease-resistant (PT) and disease-susceptible (Bourbon) coffee varieties across five niches: leaves (Lf), fruits (Fr), roots (Rt), rhizosphere soil (RS), and non-rhizosphere soil (NRS). (**A**) Bacterial α-diversity. (**B**) Fungal α-diversity. (**C**) PCoA of bacterial communities. (**D**) PCoA of fungal communities. (**E**) NMDS of bacterial communities. (**F**) NMDS of fungal communities. All analyses demonstrate significant differences in microbial diversity and community structure between varieties. Same letters indicate no significant difference (*p* > 0.05); different letters denote significant differences (*p* < 0.05).

In order to investigate the underlying mechanisms linking plant disease resistance and microbial community stability, bacterial and fungal communities were analyzed based on alpha diversity indices (Pielou’s evenness, species richness, and Shannon index). The results demonstrated a significant influence of ecological niches on bacterial diversity (Tukey’s HSD, *p* < 0.05) (Figure 2A). The species richness (3525 OTUs) and Shannon index (5.39 ± 2.25, maximum 6.76) in the non-rhizosphere soil (NRS) niche were significantly higher than in other ecological niches, except for the leaves of the susceptible (S) variety (*p* < 0.05). The influence of variety was observed only in non-rhizosphere soil. Specifically, the disease-resistant variety exhibited significantly higher species richness (3525 ± 895.94 OTUs, max. 4237) and Shannon index (5.39 ± 2.25, max. 6.76) in resistant non-rhizosphere soil (RNRS) compared to the disease-susceptible variety (SNRS: 1422.67 ± 234.35 OTUs; Shannon index: 2.35 ± 0.39) (Figure 2A, Table S5). Fruit (FRP) ecological niches showed the lowest microbial diversity, with richness (RFRP: 216.67 ± 95.87 OTUs; SFRP: 973.33 ± 1129.91 OTUs) and Shannon index (RFRP: 0.97 ± 0.13; SFRP: 1.42 ± 1.43) not differing significantly between varieties (*p* > 0.05). Notably, all three indices revealed that both varieties exhibited higher values in leaf niches than in fruit niches (Figure 2A, Table S5). Analysis of fungal diversity (Kruskal–Wallis test, *p* < 0.05) showed that plant tissue niches harbored greater diversity (Figure 2B). Specifically, roots of disease-resistant varieties (RRTS) exhibited significantly higher species richness (1473 ± 163 OTUs) and Shannon index (4.91 ± 0.31) compared to other ecological niches (*p* < 0.005). Conversely, susceptible root systems (SRTS) showed lower species richness (1234 ± 41 OTUs) and Shannon index (4.22 ± 0.21) (Figure 2B, Table S6).

### 3.3. Beta Diversity Analysis Reveals the Co-Shaping of Microbial Community Structure by Niche and Species

The objective of this study is to assess differences in bacterial and fungal community composition between susceptible (S) and resistant (R) coffee varieties across various ecological niches, including fruit (RFP), leaf (FL), root (RTS), rhizosphere soil (RS), and non-rhizosphere soil (NRS). Non-metric multidimensional scaling (NMDS) and principal coordinate analysis (PCoA) were performed using the Bray–Curtis distance metric. These analyses were supplemented by analysis of similarity (ANOSIM) and permutation multivariate analysis of variance (PERMANOVA) to test for statistically significant differences (refer to Figure 2C–E). For bacterial communities, NMDS results (stress = 0.062 < 0.1, indicating good dimensionality reduction) showed that samples were distinctly separated along the NMDS1 axis (Figure 2E), indicating that ecological niche was the primary determinant of sample distribution. Specifically, SRS, SFL, RNRS, and RFL were distributed on the right side of the NMDS1 axis, while the remaining samples were primarily on the left. Different varieties were more distinct in certain ecological niches (e.g., FL and NRS), evident from the separation between disease-resistant (R) and susceptible (S) samples within the same niche (e.g., RFL vs. SFL, RRS vs. SRS). ANOSIM analysis confirmed significant structural differences (Global R = 0.702, *p* = 0.001). PCoA results (Figure 2C) aligned with NMDS, with the PCo1 axis (explaining 61.45% of variation) mainly capturing niche-based differences, while the PCo2 axis (14.3%) potentially reflected varietal differences.

In fungal communities, the NMDS stress value was 0.081 (<0.1), indicating a reliable dimensionality reduction and clear sample separation (Figure 2F). Like bacteria, ecological niches served as primary differentiators. Notably, the rhizosphere soil of susceptible varieties (SRS) was distinctly separated from other samples (including RRS), suggesting a unique fungal community composition. Unlike bacterial communities, niche-based separation was even more distinct in fungi. ANOSIM further supported significant community differences (Global R = 0.960, *p* = 0.001). PCoA results (Figure 2D) showed that the PCo1 and PCo2 axes accounted for 41.03% and 15.22% of variation, respectively, jointly distinguishing samples by niche and variety. PERMANOVA confirmed that both ecological niche and variety significantly influenced fungal community structure (*p* = 0.001), with niche explaining the majority of variation (R^2^ = 0.8563).

This study employed NMDS, PCoA, and PERMANOVA to demonstrate that ecological niche is the predominant factor affecting microbial (bacterial and fungal) community variation in *Coffea arabica* varieties, accounting for over 85% of the observed differences. Communities in plant tissues (FRP, FL, RTS) were clearly distinguishable from those in soil (RS, NRS), particularly among fungal assemblages. Although varietal resistance (susceptibility vs. resistance) significantly influenced microbial structure (PERMANOVA *p* = 0.001), its effect size was relatively small and niche-dependent. In bacterial communities, varietal differences were most pronounced in leaves and rhizosphere soils. In fungal communities, the rhizosphere soil (SRS) of susceptible varieties exhibited a distinct composition.

### 3.4. Characteristics of Microbial Community Composition

At the genus level (Figure 1G, Tables S2, S4, S7 and S8), dominant genera were taxonomically diverse. However, a significant portion of the microbial community in plant tissues (FRP: >90%; FL/RTS: 77–91%) and soil (59–88%) was classified as “Others”, representing unclassified or low-abundance genera. These groups are likely the result of limitations in current taxonomic resolution, and their high relative abundance in some samples should be interpreted with caution. As such, no strong conclusions should be drawn from these unclassified genera.

The highest abundance of *g_unidentified_Rickettsiales* was observed in leaf tissues, particularly in resistant varieties (RFL: 22.02%) compared to susceptible ones (SFL: 17.04%). Similarly, in fruits (RFP), *g_unidentified_Rickettsiales* was more abundant in susceptible varieties (SFRP: 9.39%) compared to resistant ones (RFRP: 6.85%). However, the lack of taxonomic resolution for these genera suggests that their exact role in plant resistance or susceptibility remains unclear. The high abundance of these unclassified groups could be due to their low taxonomic identification or incomplete database coverage, rather than being biologically significant indicators of plant health or disease. Regardless of varietal resistance, most other dominant genera exhibited lower abundance than those found in other ecological niches, further emphasizing the need for caution when interpreting the functional role of unidentified or low-abundance genera.

In soil, *g_Pseudomonas* (Pseudomonas genus) showed a higher abundance in susceptible varieties (SRS: 26%, SNRS: 35%) than in resistant ones (RRS: 8%, RNRS: 0%). This pattern suggests a potential link between the presence of Pseudomonas and disease susceptibility, as some Pseudomonas species are known plant pathogens that may exacerbate plant disease by producing toxins or promoting pathogenic conditions.

At the phylum level of fungal communities, Ascomycota was the predominant phylum across all ecological niches and varieties, with relative abundances ranging from 53.89% to 75.44% (Figure 1H). Basidiomycota was the second-most abundant phylum, with its highest abundance detected in infected leaf samples (SFL: 31.07%). At the genus level (Figure 1H), *Neurospora* was significantly enriched in infected soils (SNRS: 21.27%; SRS: 17.19%) but exhibited low abundance in other niches. This suggests that *Neurospora* may be involved in disease progression and soil degradation processes associated with plant infection. Interestingly, *Neurospora* and *Zymoseptoria* showed elevated abundance in plant tissues of resistant varieties (RFRP: 15.37%, RFL: 6.95%, RRTS: 4%) compared to susceptible varieties (SFRP: 9.48%, SFL: 3.62%), indicating a potential beneficial role or adaptation to plant resistance mechanisms.

Mortierella was more abundant in the SNRS niche (7.18%), potentially indicating its role in soil health and microbial competition in the rhizosphere of susceptible varieties. Community composition analyses using phylogenetic trees (Figure S2) revealed that fungal communities (A) were primarily clustered by niche type—i.e., above-ground (RFP/FL) vs. below-ground (RTS/RS/NRS). SNRS samples formed a distinct cluster enriched with c_Sordariomycetes, indicating a fungal community significantly different from those of other groups. RRS samples were more similar to above-ground samples (RFP/FL), suggesting overlapping community structures. In bacterial communities (B), samples were grouped into two major clusters: plant tissue-associated communities (including both endophytic and epiphytic bacteria, RFP/FL/RTS) and environmental soil communities (RS/NRS). Within soil clusters, rhizosphere soil (RS) and non-rhizosphere soil (NRS) showed a clear trend of separation.

### 3.5. Changes in Dominant Species Markers of Microbial Communities and Taxonomic Level Analysis

**Figure 3:**
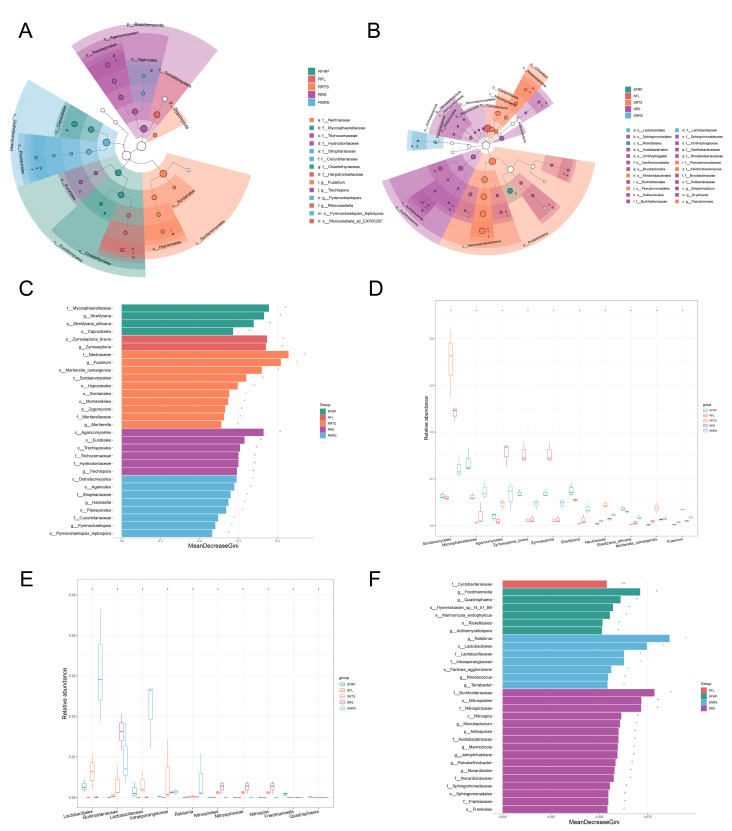
Analysis of 16S bacterial microbial community composition and characteristics in disease-resistant PT varieties and disease-susceptible Bourbon varieties. Note: Analysis of bacterial community composition and feature importance in disease-resistant (PT) and susceptible (Bourbon) varieties across niches (Lf, Fr, Rt, RS, NRS). (**A**,**B**) Bacterial classification trees for PT (**A**) and Bourbon (**B**). (**C**) Random forest feature importance (PT). (**D**) Distribution of key bacterial features in PT niches. (**E**) Random forest feature importance (Bourbon). (**F**) Distribution of key bacterial features in Bourbon niches.

**Figure 4:**
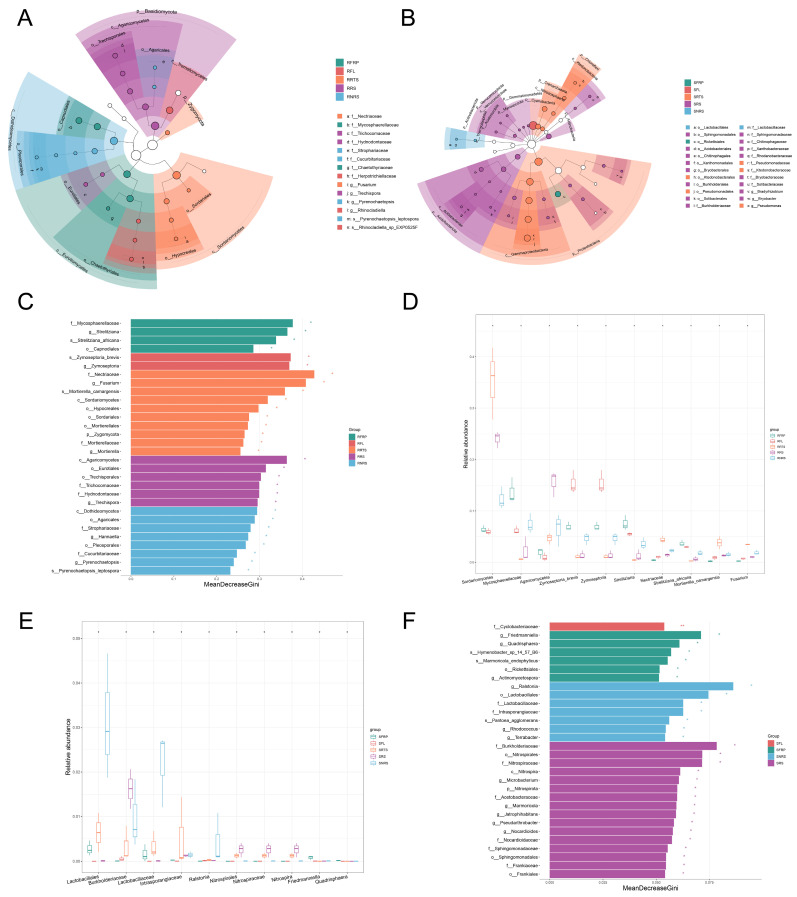
Analysis of fungal microbial community composition and characteristics in disease-resistant PT varieties and disease-susceptible Bourbon varieties. Note: Analysis of fungal community composition and feature importance in disease-resistant (PT) and susceptible (Bourbon) varieties across niches (Lf, Fr, Rt, RS, NRS). (**A**,**B**) ITS-based fungal classification trees for PT (**A**) and Bourbon (**B**). (**C**) Random forest feature importance (PT). (**D**) Distribution of key fungal features in PT niches. (**E**) Random forest feature importance (Bourbon). (**F**) Distribution of key fungal features in Bourbon niches.

Since the Linear Discriminant Analysis (LDA) values were not log-transformed, this study did not apply a predefined LDA threshold. Instead, microbial groups exhibiting significant differences were identified based on a significance level of *p* < 0.05. These were further ranked by integrating LDA values with feature importance scores derived from the random forest algorithm (Mean Decrease Gini), providing a comprehensive assessment of potential microbial biomarkers (Figure 3 and Figure 4).

Different microbial biomarkers were identified between susceptible (S) and resistant (R) coffee varieties across various ecological niches (Figure 3). In plant tissues (leaves and roots) of resistant varieties, there was a pronounced enrichment of the Cyanobacteria phylum (*p_Cyanobacteria*), along with Chloroflexi and Myxococcota. In contrast, rhizosphere and non-rhizosphere soils associated with resistant varieties were enriched in Actinobacteriota and Proteobacteria. Notably, Proteobacteria also dominated in disease-resistant fruits (RFRP).

Regarding fungal communities (Figure 4), rhizosphere soils (RRS) of resistant varieties were enriched in the Basidiomycota phylum, while root tissues (RRTS) exhibited significant abundance of the Zygomycota phylum. Furthermore, specific fungal species were enriched in above-ground tissues: *s_Zymoseptoria* brevis in leaves (RFL) and *s_Strelitziana africana* in fruits (RFRP).

In contrast, the rhizosphere soil (SRS) of susceptible varieties displayed marked enrichment of *p_Acidobacteriota* and *p_Verrucomicrobiota*, while roots (SRTS) were enriched with *p_Crenarchaeota*, serving as a diagnostic microbial signature. Leaves (SFL) of susceptible varieties showed high abundance of *p_Cyanobacteria* and significant enrichment of the Cyclobacteriaceae family (*f_Cyclobacteriaceae*, *p* < 0.001) (Figure 3).

Within fungal communities (Figure 4), the Ascomycota phylum accounted for up to 75% of the total relative abundance. Diseased leaf tissues exhibited reduced fungal diversity, with dominant groups constituting only ~4%, suggesting a highly skewed (monocotrophic) community structure. Enrichment of *s_Cryptococcus heimaeyensis* and *s_Oidiodendron maius* in fruits (SFRP) and non-rhizosphere soil (SNRS), respectively, further highlights the distinctive microbial characteristics associated with susceptible varieties.

### 3.6. Network Analysis of Microbial Co-Occurrence Patterns in Coffee-Associated Ecological Niches

**Figure 5:**
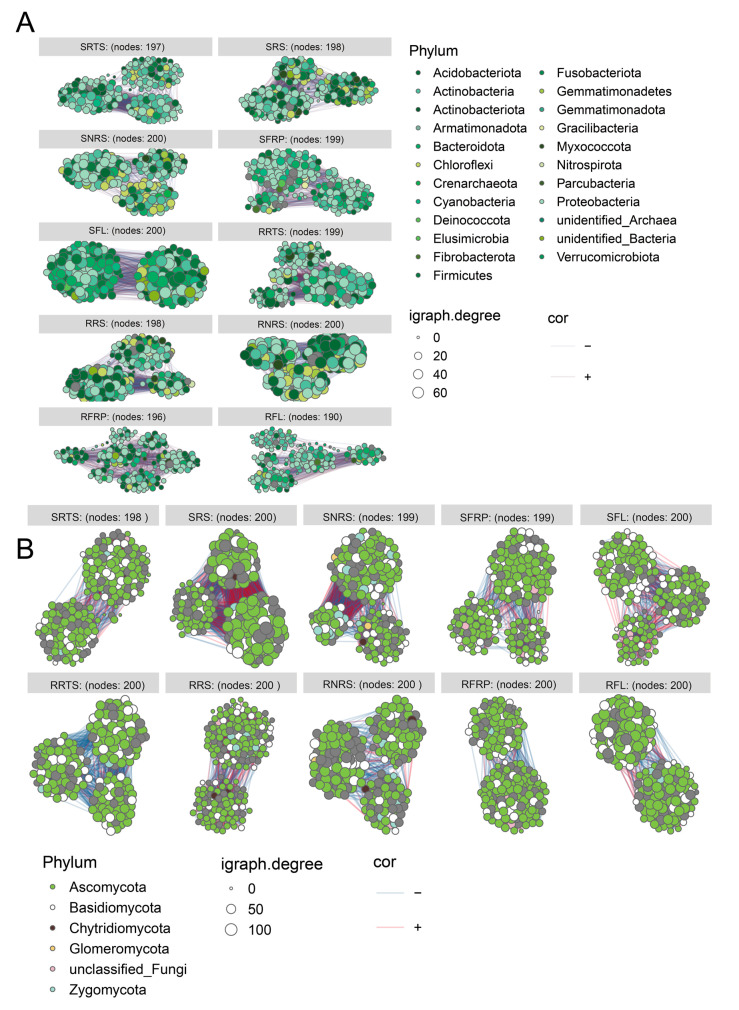
Microbial co-occurrence network analysis using the ggClusterNet package. Note: (**A**) Bacterial network: Each node represents a bacterial genus, with the node size corresponding to the degree (i.e., the number of connections). The color of the edges indicates the correlation between nodes: positive correlations are shown in red, while negative correlations are shown in blue. (**B**) Fungal network: each node represents a fungal genus, with the same visualization principles as in panel A applied.

This study systematically compared the microbial community interaction patterns of disease-resistant (R) and disease-susceptible (S) coffee varieties across diverse ecological niches. Microbial co-occurrence networks were analyzed in terms of structural properties, topological roles, and random network benchmarks (Figure 5 and Figure S3). The results revealed that bacterial networks in resistant varieties exhibited significantly higher modularity (modularity index > 0), indicating a more compartmentalized and functionally diversified structure (*p* < 0.05). In contrast, the fungal networks of resistant varieties showed low centrality and high redundancy, suggesting enhanced stability through distributed interactions and functional overlap.

In fungal networks, node-level analysis in the rhizosphere (RS) indicated that susceptible varieties had significantly higher average degree centrality than resistant ones (*p* = 0.008), pointing to a more concentrated yet potentially less robust network. Resistant varieties displayed a long-tail distribution in fungal network connectivity across ecological niches, indicative of stronger decentralization and modular independence.

Topological role analysis using Zi-Pi plots (Figure S4) further showed that disease-resistant varieties were enriched with cross-module hub nodes—both module hubs and connectors. Notable examples include Proteobacteria OTU_640 (Zi > 2.5, Pi > 0.62) and Actinobacteria OTU_2764 in the bacterial networks, and Ascomycota OTU_78 and Basidiomycota OTU_5 in fungal networks.

To further validate network characteristics, observed networks were compared with randomized counterparts generated using the Erdős–Rényi model (Figure S4C,D). Real microbial networks exhibited significantly higher mean clustering coefficients compared to random networks (*p* < 0.001), and their degree distributions followed power-law or right-skewed patterns, confirming non-random, small-world properties.

Above-ground niches (SFL, SFRP) featured real networks with high local concentration and extended tail distributions, indicating the presence of locally dominant, highly connected nodes. Subterranean niches such as SNRS and RRTS demonstrated pronounced long-tail degree distributions, reflecting the presence of multiple central nodes and complex co-occurrence interactions.

### 3.7. Functional Prediction of Bacterial and Fungal Communities Exhibiting Varied Resistance Traits

**Figure 6:**
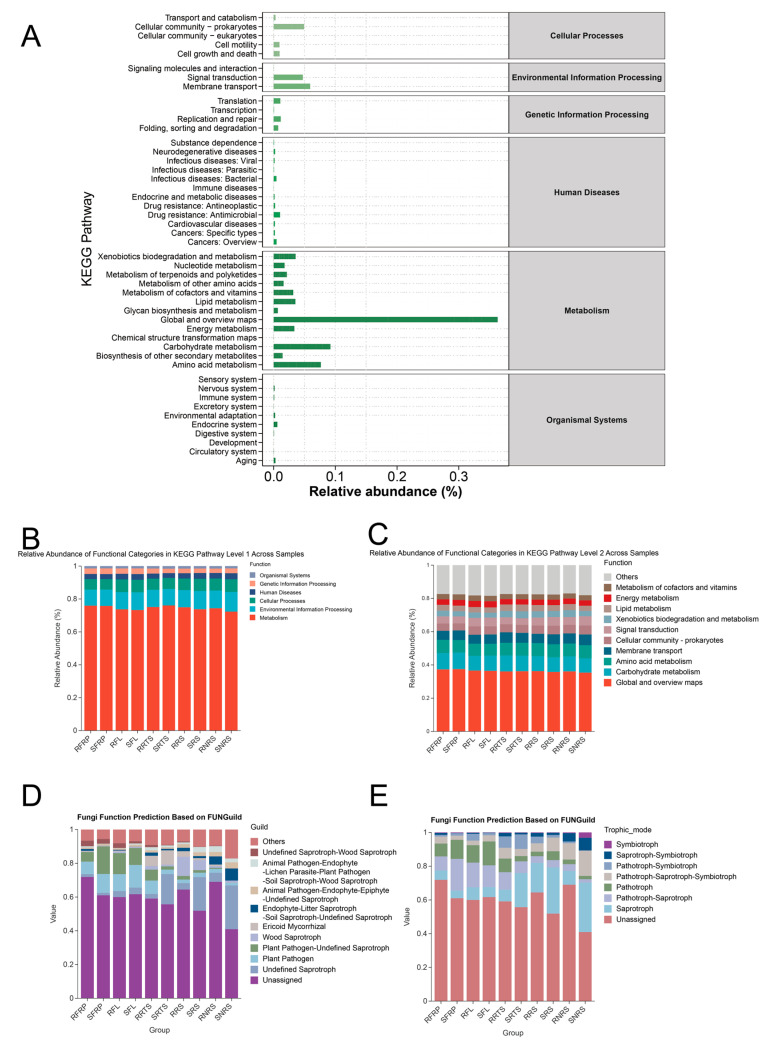
Functional prediction of bacteria and fungi. Note: (**A**–**C**) Bacterial functional prediction based on the Tax4Fun database: shows the relative abundance (%) of different groups across various KEGG pathways. (**D**) Fungal functional prediction based on the FUNGuild database: displays the distribution of different fungal taxa across groups. (**E**) Fungal functional prediction based on the FUNGuild database: illustrates the distribution of nutritional modes (e.g., symbiotic, saprophytic) across different groups.

To investigate functional differences in bacterial communities among plant varieties, we employed KEGG (Kyoto Encyclopedia of Genes and Genomes) pathway prediction based on 16S rRNA gene sequences using the Tax4Fun database (Figure 6A–C). The analysis revealed that metabolism-related pathways were predominant across all samples, with the highest abundance observed in the “Global and overview maps” category (Figure 6A). Notable differences were identified in specific pathways associated with plant–microbe interactions. Carbohydrate and amino acid metabolism pathways were enriched, suggesting the involvement of bacterial communities in utilizing plant root exudates and organic matter decomposition. Additionally, complex signal transduction pathways were detected, indicating bacterial mechanisms for environmental signal perception and response. The distribution of metabolic functions was relatively balanced across different groups (Figure 6B,C). Genes associated with drug resistance were also identified in some samples, indicating the presence of resistance genes in the soil.

To further explore the ecological roles of fungal communities, we classified fungal taxa into nutritional modes using the FUNGuild database (Figure 6D,E). Although some fungal functions remained unassigned, significant functional divergence was observed between disease-resistant (PT varieties) and disease-susceptible (Bourbon variety) plants. Specifically, reciprocal patterns were identified between pathotrophic and saprotrophic functional types. Pathotrophs, particularly plant pathogens, were substantially enriched in fruit (SFRP) and leaf (SFL) tissues of susceptible varieties, whereas saprotrophs, such as wood-decaying fungi, dominated the rhizosphere soil of resistant varieties.

## 4. Discussion

This study utilized high-throughput sequencing technology to systematically compare the bacterial and fungal communities of two coffee varieties—one resistant to coffee leaf rust (PT) and one susceptible (Bourbon variety)—across five key ecological niches: fruit, leaves, roots, rhizosphere soil, and non-rhizosphere soil. The results clearly revealed that the two coffee plant cultivars exhibit significant differences in microbiome community structure, species composition, network interaction patterns, and predicted functions. These findings suggest that the microbiome does not simply respond passively to the host but rather plays an active role in shaping plant health phenotypes. The distinct characteristics of the microbiome may serve as a crucial biological foundation for determining the disease resistance of coffee varieties. An interaction effect between niche and variety was also evident, suggesting that varietal influence on microbial communities depends on specific ecological contexts.

### 4.1. Dual Driving Forces of Ecological Niche and Host Genotype

Microbial composition in plant organs is influenced by a variety of biotic and abiotic factors, including soil chemistry, environmental conditions, and plant genotype [25]. Soil pH, salinity, and structure are particularly important for below-ground microbiota, while climate, pathogen presence, and human practices affect both above- and below-ground microbial communities, with root morphology and exudates playing a key role in microbial recruitment [25]. An ecological niche can be conceptualized as a multidimensional entity, with each dimension representing the abiotic conditions or biotic resources required for a species to thrive [41]. This view aligns with the findings of numerous microbiome studies [42,43]. The present study confirms that ecological niches are the primary drivers shaping the structure of coffee microbiome communities (Figure 1 and Figure 2). There is clear compartmentalization between the microbial communities of coffee plant tissues (endophytic microbes) and soil (rhizosphere and non-rhizosphere), reflecting the substantial differences in nutrient availability, physical–chemical conditions, and host immune pressures across different habitats. However, the significant discovery of this study is that coffee plant resistance genotypes may impose ecologically specific selective pressures on microbial communities (Figure 2). This host-mediated selective effect appears to lead to significant differentiation between disease-resistant and susceptible varieties in microbial diversity, the abundance of key taxa, and community stability across specific ecological niches. These findings are consistent with observations in other crops, such as soybeans and chili peppers, where plant genotypes have been identified as crucial factors in shaping beneficial microbiomes [44,45]. Both below-ground and above-ground plant-associated bacteria have been shown to enhance host resistance against pathogen infection either through commensal–pathogen interactions or through modulating plant defense [25].

### 4.2. Microbiome Characteristics of Disease-Resistant Varieties: A Stable, Diverse, and Functionally Redundant “Defence Alliance”

The interactions between plants and microorganisms are crucial for plant health, with specific microorganisms playing a pivotal role in the growth and fruit development of coffee plants [30]. Qiu et al. analyzed the soil microbial compositions in the rhizospheres of wilt-resistant and susceptible melon varieties to screen for bio-control soil microorganisms that could prevent melon wilt [46]. In a recent study, Ketehouli et al. applied a soil microbiome transplant (SMT) to restore the microbiome balance, which potentially reduced the severity of leaf diseases [47]. In a study of wheat, the inoculation of the rhizosphere microbiome of resistant cultivars suppressed pathogen infection and enhanced plant growth, indicating that wheat resistance to soil-borne virus disease depended on the interaction of the host with the microbial community around it [48]. To screen for biological control microorganisms that could prevent CLR, it is necessary to conduct a comprehensive analysis of microbial community composition, structure, and network interactions within the ecological niches of disease-resistant and susceptible varieties. The microbiome of disease-resistant PT varieties has been shown to exhibit typical “healthy” characteristics. In key ecological niches such as non-rhizosphere soil (RNRS), bacterial communities in these varieties show higher α-diversity (Figure 2A,B; Tables S5 and S6). This higher diversity is typically associated with stronger ecosystem functions and increased stress resistance [49]. Earlier research employing LEfSe analysis identified significant metagenomic biomarkers in fungal and bacterial communities from soil and fruit samples across six coffee genotypes. In this study, LEfSe analysis revealed that disease-resistant varieties were enriched with beneficial bacterial groups, such as the Actinobacteriota phylum in soil, which is known for antibiotic production and plays a crucial role in biological control [50] (Figure 3). This was further corroborated by functional predictions, which indicated that the rhizosphere soil (RRS) of disease-resistant varieties was significantly enriched with saprotrophic (Saprotroph) fungi. These fungi decompose organic matter efficiently, competing with pathogens for ecological niches and nutrients, thus establishing a “pathogen-suppressing soil”—a key mechanism of plant disease resistance. Metatranscriptomics, functional studies, or labeling of carbon absorption revealed that overrepresentation of specific fungal phyla in the rhizosphere correlates with their increased activity around the roots or services they provide to the host plants [51].

The interactions among microorganisms are crucial in determining the structure and function of microbial communities. The use of microbial co-occurrence networks in specific environments can provide deeper insights into the structural characteristics and complexity of these systems [52,53,54]. For example, prior studies combining microbial ecology and phylogenetic analysis with structural equation modeling have linked microbial community characteristics to denitrification rates, underscoring the importance of understanding microbial communities for effective ecosystem management [52]. In this study, the R ggClisterNet package was employed to visualize microbial co-occurrence networks across various coffee ecological niches for CLR-resistant varieties [40] (Figure 5, Figures S3 and S4). Microbial network analysis revealed that disease-resistant varieties promote more robust and functionally redundant microbiomes by increasing modularity, decentralizing network topology, and enriching cross-module hubs (e.g., OTU_640 and OTU_78). In contrast, susceptible varieties exhibited denser but less structurally heterogeneous networks, which may compromise their resistance to environmental fluctuations and pathogen pressure. These network features offer mechanistic insights into the microbial ecological foundations of host plant disease resistance. Notably, the co-occurrence networks of disease-resistant varieties exhibited higher modularity, more complex structures, and greater stability. These networks contained numerous key “connector” nodes—such as OTUs from the Ascomycota, Proteobacteria, and Actinomycota phyla—which connect different functional modules, significantly enhancing network redundancy and resilience to disturbances (e.g., pathogen invasion) (Figure S4A). The analysis of Zi-Pi plots (Figure S4) reveals that disease-resistant varieties are enriched with cross-module hub nodes, which are essential for maintaining ecological stability and functional redundancy. In contrast, susceptible varieties show a predominance of peripheral nodes, particularly in below-ground environments, indicating a lack of resilience and increased vulnerability to pathogen invasion. These findings highlight the structural differences between resistant and susceptible plant varieties in microbial network organization. Identification of key network elements, in this case modules or hubs, may facilitate practical application of microbial networks to modern agriculture [55]. Similar to findings observed in common bean cultivars with varying degrees of Fox resistance [56], our results suggest that breeding for disease resistance may alter the microbial network composition. In particular, resistant varieties appear to harbor microbial communities that support resilience, as reflected by the enrichment of cross-module hub nodes, which contrasts with the peripheral nodes found in susceptible varieties. This stable, interconnected network structure suggests that disease-resistant varieties have effectively “recruited and maintained” a microbial defense alliance that exhibits synergistic effects. For example, Penicillium (genus), found in the Ascomycota phylum, was identified as a potentially beneficial microorganism for coffee plants [57]. Additionally, Aspergillus and Penicillium within the Ascomycota group were enriched in the rhizosphere soil (RRS) and root system (RRTS) of disease-resistant varieties. While these genera are the most prevalent toxin-producing fungi in coffee, diminishing final beverage quality [58], the microbial defense alliances in resistant varieties allow them to thrive under CLR conditions. However, further efforts are needed to improve the quality of these varieties by targeting these genera, thereby enhancing their beneficial properties.

### 4.3. Microbiome Characteristics of Disease-Susceptible Varieties: Ecological Imbalance, Pathogen Enrichment, and Fragile Networks

Previous studies have demonstrated that soil microorganisms are also present on coffee fruits, transferring between them [59]. Additionally, coffee fruit peel and litter have been identified as primary drivers of bacterial community diversity and richness, as well as shifts in bacterial community structure and function [60]. Consequently, the cultivation and management of coffee plants are particularly susceptible to ecological imbalances. In stark contrast to disease-resistant varieties, the microbiome of disease-susceptible Bourbon varieties exhibits multiple signs of “imbalance”. Functional predictions indicate that the above-ground parts of susceptible varieties (e.g., fruit SFRP, leaf SFL) are significantly enriched with pathotrophic fungi, which suggests higher pathogen infection pressure (Figure 6D,E).

Earlier research has identified unique microbial characteristics in Bourbon and Castillo varieties, highlighting the presence of diverse microorganisms, including uncultivable bacterial species, in soil and vegetation layers. These studies also suggested that microbial communities might be genotype-dependent [31]. A particularly striking observation in this study was the explosive enrichment of the *Neurospora* genus in both rhizosphere (SRS) and non-rhizosphere soil (SNRS) of susceptible varieties. While *Neurospora* is not typically considered a major plant pathogen, its rapid growth as an opportunistic fungus often indicates microbial ecological imbalance [61]. Additionally, the high abundance of *Pseudomonas* in the soil of susceptible varieties may point to a weakened root defense capacity, as *Pseudomonas* species, although commonly *Pseudomonas* is present in high numbers, it might suggest *Neurospora*, to thrive. The resulting ecological imbalance creates favorable conditions for the establishment and infection of true pathogens, such as the soil-borne stage of coffee leaf rust and other root pathogens.

Interestingly, although Pseudomonas is known to include numerous opportunistic plant pathogens, many species within this genus are also recognized for promoting plant growth by inhibiting pathogens, synthesizing growth-stimulating hormones, and enhancing plant resistance [62]. From a network structure perspective, the microbial networks of susceptible varieties exhibit high connectivity but low modularity and lack core hub nodes, resulting in a fragile network configuration (Figure 5, Figures S3 and S4). Such networks are more prone to cascading failures when subjected to external disturbances and are unable to form effective synergistic defenses. This indicates that susceptible varieties are unable to recruit beneficial microbial communities and lack resilience within their microbial networks, making them more vulnerable to pathogen threats. This study systematically reveals significant differences in the structure and function of microbiomes across multiple ecological niches between disease-resistant and susceptible coffee varieties. It underscores that coffee disease resistance is not solely determined by the plant’s own genes but is a macro-level manifestation of plant–microbe interactions, or the “plant holobiont” [63,64]. Disease-resistant varieties appear to enhance their defense mechanisms through the recruitment of beneficial microbial communities, the establishment of a pathogen-suppressing micro-environment dominated by saprophytic bacteria, and the maintenance of a stable, modular microbial network. Similar to studies on disease-resistant wheat cultivars, coffee disease-resistant varieties also enhance their disease resistance by maintaining higher microbial diversity and specific microbial community structures. In these varieties, changes in the rhizosphere microbiome are closely associated with plant health, with certain beneficial microorganisms, such as Pseudomonas, playing a key role in plant growth and disease resistance [48]. In contrast, the microbiomes of susceptible varieties show signs of pathogen enrichment, ecological imbalance, and network fragility. As mentioned in research on the genetic basis of microbiome recruitment in grapevine, grapevine genetics substantially shape microbial community recruitment even under varying environmental conditions [65].

This study offers novel insights into the synergistic plant–microbe disease resistance mechanism and provides a framework for future research. To further validate the roles of key differentially abundant species in coffee disease resistance, future studies should employ isolation and re-inoculation experiments. Multi-omics integration, combining metagenomics, metatranscriptomics, and metabolomics, can provide a comprehensive understanding of the functional potential of these microorganisms (e.g., antibiotic synthesis genes and pathogenic factors) and their expression activity within the host. This will reveal the molecular mechanisms behind plant–microbe interactions. Furthermore, the findings of this study suggest that future research could explore constructing a “synthetic microbiota” composed of core microorganisms from disease-resistant varieties, with the goal of applying it to susceptible varieties. This approach, through microbiota transplantation or regulation, could enhance coffee disease resistance and provide a scientific basis for developing new biological control strategies. However, it is important to note that the conclusions drawn from this study are based on correlative data, and further functional validation studies are needed to confirm the causal roles of these microbial species in coffee disease resistance.

## 5. Conclusions

This study suggests that the resistance of coffee varieties to leaf rust may be closely linked to the structure, function, and network stability of their multi-ecological niche microbiomes. The results reveal a clear binary opposition in disease resistance at the microbial level. Disease-resistant varieties (PT) form a “synergistic defense system” by enriching beneficial microbial communities (e.g., *Actinomycetes*), establishing a disease-suppressing microenvironment dominated by saprophytic bacteria, and maintaining a highly modular and stable microbial network. In contrast, the microbiome of susceptible varieties (e.g., Bourbon variety) shows an enrichment of pathogen-associated nutritional types and stress indicator bacteria (such as the genus Rhizopus), as well as a fragile and easily disrupted interaction network—collectively indicating an “ecological imbalance”. This finding emphasizes that plant disease resistance is not solely determined by the plant’s own genes but is a macro-level manifestation of the interaction between the plant and its microbiome. Consequently, future research should focus on screening and utilizing core beneficial microorganisms from disease-resistant varieties to develop novel microbial agents. These agents could enhance disease resistance by modifying the rhizosphere or foliar microecology of susceptible varieties, providing new biological control strategies for the sustainable development of the coffee industry.

## Data Availability

The raw 16S rRNA and ITS sequencing data generated in this study have been deposited in the NCBI Sequence Read Archive (SRA) under BioProject accession number PRJNA1298541. All other data supporting the findings are included within the article and its Supplementary Materials.

## Supplementary Materials

The following supporting information can be downloaded at: https://www.mdpi.com/article/10.3390/microorganisms13081909/s1, Figure S1: Ecological Niche Diversity Curves for Different Resistant Varieties of Coffea arabica; Figure S2 Microbial Community Structure Analysis: Distribution of Fungal and Bacterial Communities in Different Ecological Niches; Figure S3 Robustness, Negative Correlation Proportions, and Module Similarity in Microbial Co-occurrence Network Analysis; Figure S4 Microbial Co-occurrence Network Analysis for Bacteria and Fungi; Table S1: 16S rRNA Gene Data Preprocessing Statistics and Quality Control Analysis of Disease-Resistant Cultivars of *Coffea arabica*; Table S2: OTU Annotation Results of 16S rRNA Gene in Disease-Resistant Cultivars of *Coffea arabica*; Table S3: ITS Data Preprocessing Statistics and Quality Control Analysis of Disease-Resistant Cultivars of *Coffea arabica*; Table S4: ITS-OTU Annotation Results of Disease-Resistant Cultivars of *Coffea arabica*; Table S5: Bacterial Alpha Diversity Analysis of Disease-Resistant Cultivars of *Coffea arabica*; Table S6: Fungal Alpha Diversity Analysis of Disease-Resistant Cultivars of *Coffea arabica*. Table S7: Summary of Taxonomic Classification and Unclassified OTU Proportions in Bacterial Communities. Table S8: Summary of Taxonomic Classification and Unclassified OTU Proportions in Fungal Communities.
