# Supplementary material for: The Role of Coffee Microbiomes in Pathogen Resistance Across Varieties and Ecological Niches
Source: Microorganisms. 2025 Aug 15;13(8):1909. doi: 10.3390/microorganisms13081909 (PMC12388573; doi:10.3390/microorganisms13081909)
Supplement: Supplementary file 1 [file microorganisms-13-01909-s001.zip › Figure S.pdf]

Bioinformatics and Statistical Analysis Workflow

This study selected two Arabica coffee varieties—the susceptible Bourbon and the resistant PT—that exhibit significant differences in resistance to coffee leaf rust. Samples were collected from five ecological niches: fruits, leaves, absorbent roots, rhizosphere soil, and non-rhizosphere soil. Each variety was sampled in triplicate, yielding a total of 30 samples. Following collection, samples were promptly transported at 4°C, and total DNA was extracted using the CTAB method in the laboratory. Polymerase chain reaction (PCR) amplification was performed using specific primers targeting the V4 region of the 16S rRNA gene for bacteria and the internal transcribed spacer (ITS) region for fungi. Libraries were constructed, and high-throughput sequencing was performed using the Illumina NovaSeq 6000 platform.

Subsequently, filtration, assembly, and removal of chimeric sequences were performed, followed by clustering of the data into operational taxonomic units (OTUs) at 97% similarity. Taxonomic annotation of bacterial and fungal sequences was conducted using the Silva and Unite databases, respectively. Alpha diversity indices and beta diversity distance matrices were calculated within the R software environment, and multidimensional analyses of community structure differences were performed using PCoA, NMDS, PERMANOVA, and ANOSIM. LEfSe and random forest analyses were employed to identify microbial taxa exhibiting significant differences between groups. A microbial co-occurrence network was constructed to assess network topology characteristics and stability. Correlation analysis was applied to standardize abundance data for this purpose. This analysis was further used to explore network structural differences and key driving factors. All statistical analyses and visualizations were performed in R, ensuring systematic and reproducible data analysis.

Supplementary Information

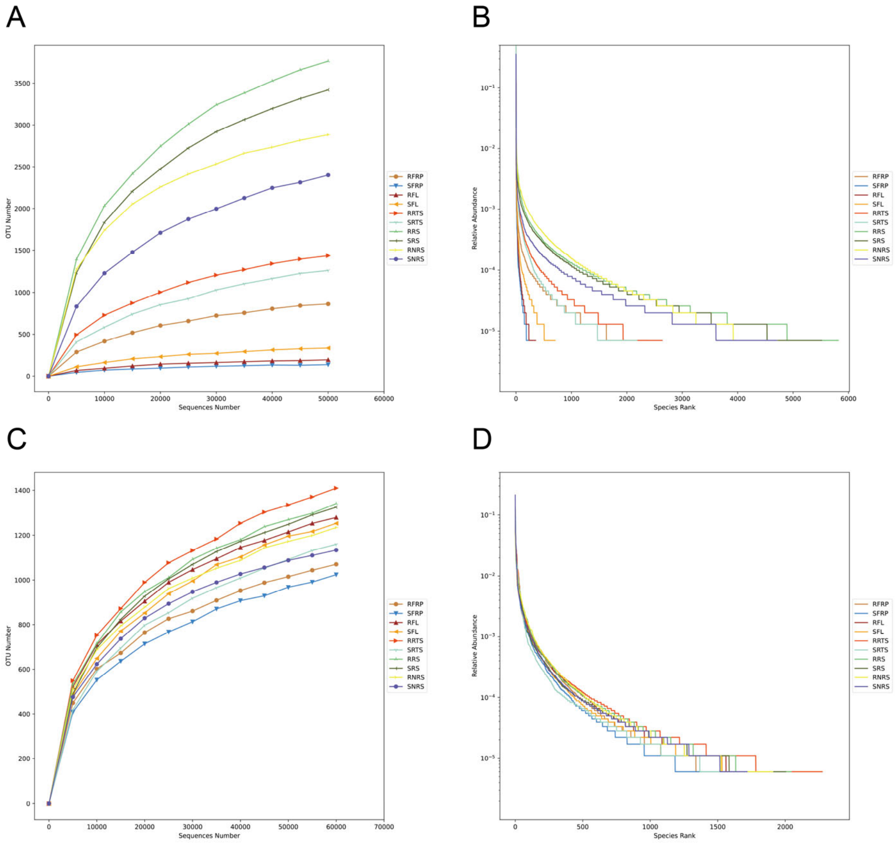

Figure S1 Ecological Niche Diversity Curves for Different Resistant Varieties of *Coffea arabica*

Note: A Grouping dilution curves based on bacterial OTUs; B Rank abundance curves for each sample based on OTUs; C Grouping dilution curves based on fungal OTUs; D Rank abundance curves for each sample based on OTUs.

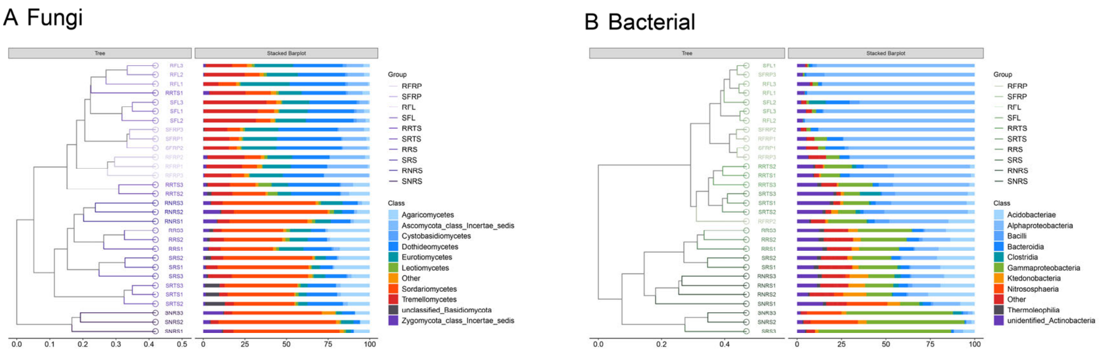

Figure S2 Microbial Community Structure Analysis: Distribution of Fungal and Bacterial Communities in Different Ecological Niches

Note: Figure S2 shows the distribution of fungal and bacterial communities in different ecological niches between the disease-resistant PT variety and the disease-susceptible Bourbon variety. The communities are represented using phylogenetic trees and stacked bar charts to illustrate community composition and relative abundance.

Note: A Phylogenetic tree and stacked bar chart of fungal communities. A shows the relative abundance distribution of fungal communities in different groups (e.g., RFRP, SFRP, etc.), with a phylogenetic tree (dendrogram) illustrating the phylogenetic relationships among communities and a stacked bar chart displaying the abundance changes of various fungal groups (e.g., *Ascomycota*, *Basidiomycota*, etc.) across groups; B Phylogenetic tree and stacked bar chart of bacterial communities. B shows the abundance of bacterial communities across different groups, covering various bacterial phyla (e.g., *Acidobacteria*, *Alphaproteobacteria*, etc.). The combination of the dendrogram and stacked bar chart illustrates the distribution characteristics of various bacterial phyla across different ecological niches.

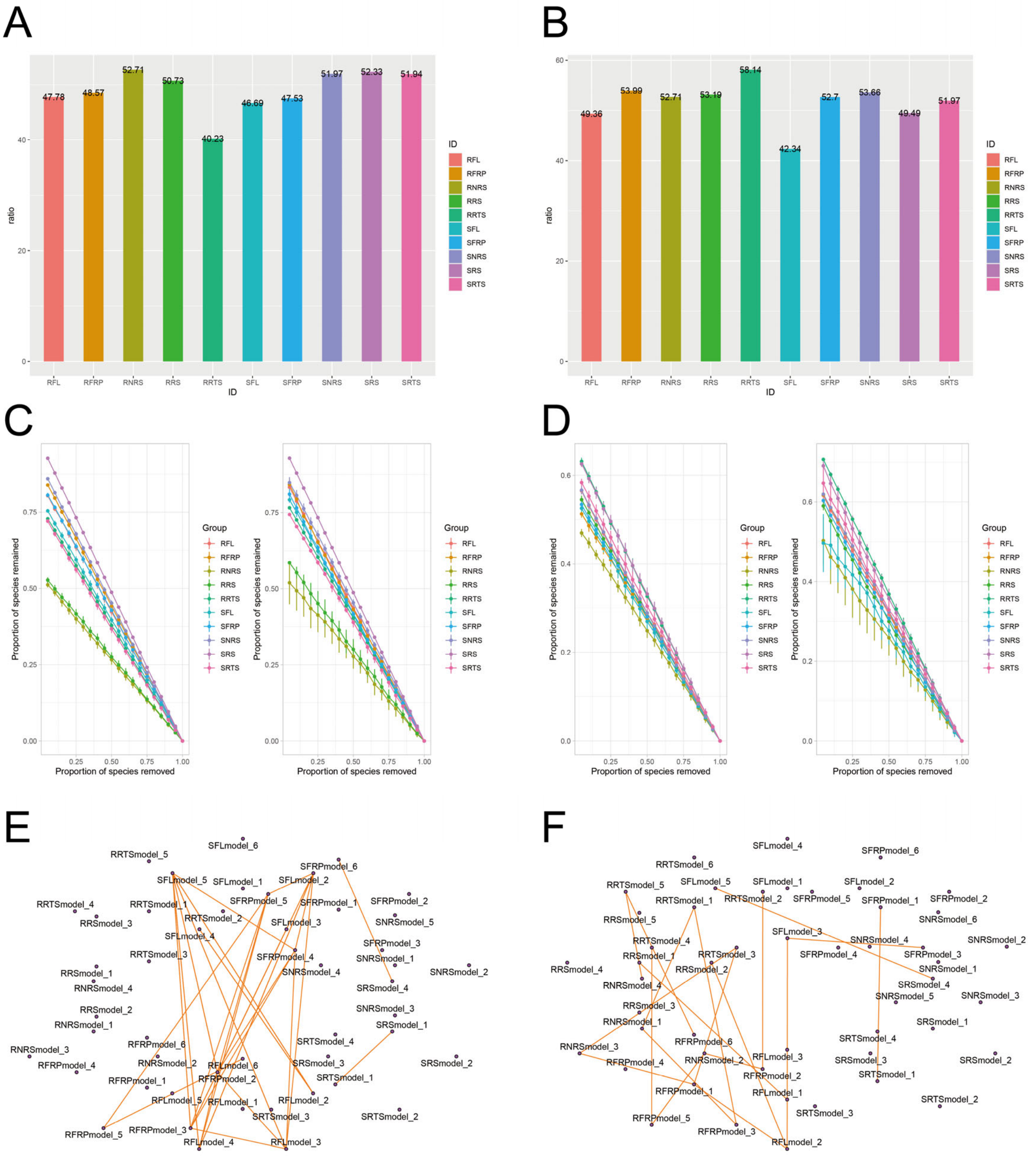

Figure S3 Robustness, Negative Correlation Proportions, and Module Similarity in Microbial Co-occurrence Network Analysis

Note: A Negative Correlation Proportions in Bacterial Microbial Co-occurrence Network Analysis across Different Groups, B Negative Correlation Proportions in Fungal Microbial Co-occurrence Network Analysis across Different Groups, C Robustness of Bacterial Microbial Co-occurrence Network

Analysis, D Robustness of Fungal Microbial Co-occurrence Network Analysis, E Module Similarity in Bacterial Microbial Co-occurrence Network Analysis, F Module Similarity in Fungal Microbial Co-occurrence Network Analysis.

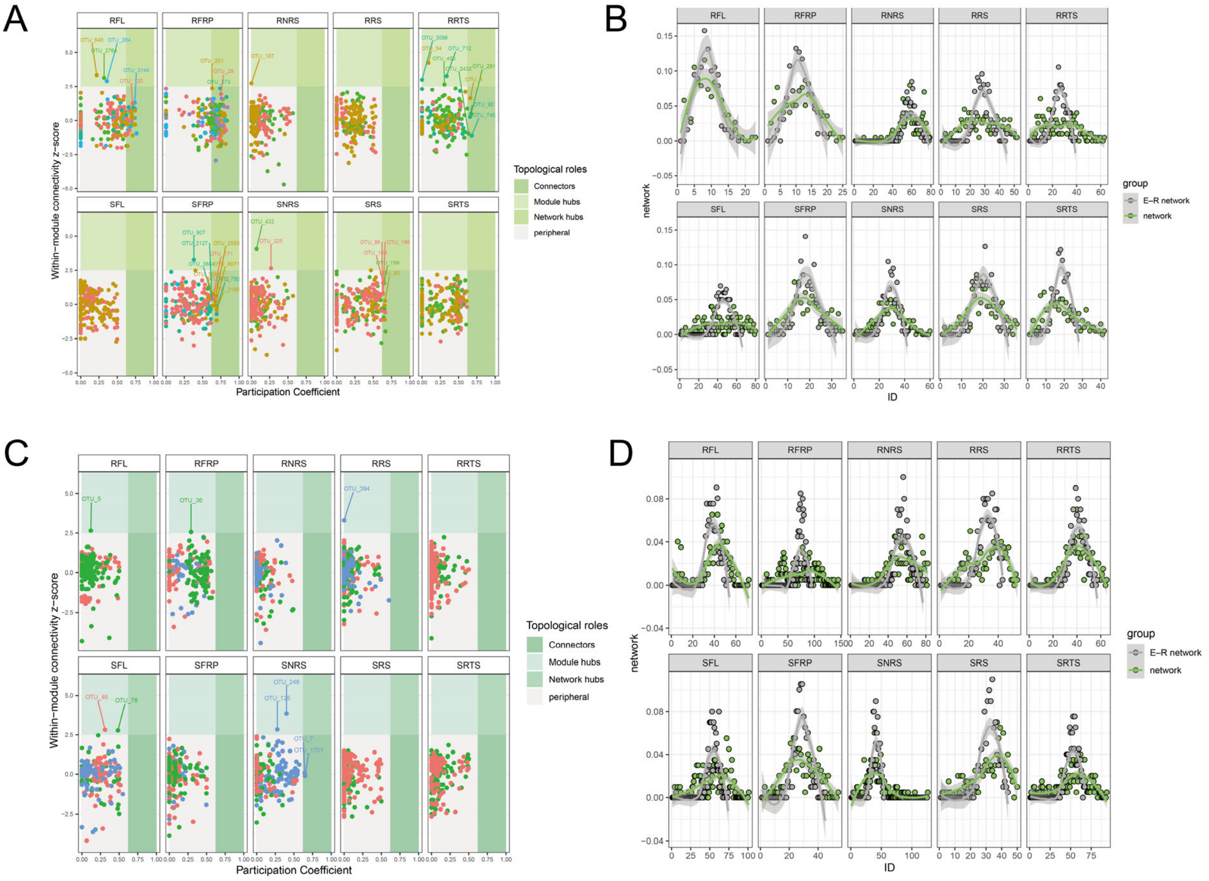

Figure S4. Microbial Co-occurrence Network Analysis for Bacteria and Fungi

Note: A Bacterial Network Topological Roles Distribution: The distribution of nodes within different topological roles in the bacterial network, including connectors, module hubs, network hubs, and peripheral nodes, based on the participation coefficient. B Bacterial Network Power-Law Distribution: The distribution of bacterial network connectivity following a power-law pattern, with the ID on the x-axis and the network degree on the y-axis. C Fungal Network Topological Roles Distribution: The distribution of nodes within different topological roles in the fungal network, including connectors, module hubs, network hubs, and peripheral nodes, based on the participation coefficient. D Fungal Network Power-Law Distribution: The distribution of fungal network connectivity following a power-law pattern, with the ID on the x-axis and the network degree on the y-axis.
